# Fatty acid transporter protein isoform 2 (FATP2) inhibitor lipofermata inhibits Zika virus replication and blocks fatty acid uptake in SH-SY5Y neuroblastoma cell line

**DOI:** 10.1128/spectrum.02140-25

**Published:** 2026-03-11

**Authors:** Songkran Thongon, Chompunuch Boonarkart, Thanyaporn Sirihongthong, Kunlakanya Jitobaom, Kessiri Kongmanas, Prasert Auewarakul

**Affiliations:** 1 Graduate Program in Molecular Medicine, Faculty of Science, Mahidol University, Bangkok, Thailand; 2 Department of Microbiology, Faculty of Medicine Siriraj Hospital, Mahidol University, Bangkok, Thailand; 3 Emerging Infectious Diseases Research Unit, Research Department, Faculty of Medicine Siriraj Hospital, Mahidol University, Bangkok, Thailand; 4 Division of Dengue Hemorrhagic Fever Research, Research Department, Faculty of Medicine Siriraj Hospital, Mahidol University, Bangkok, Thailand; 5 Siriraj Center of Research Excellence in Dengue and Emerging Pathogens, Faculty of Medicine Siriraj Hospital, Mahidol University 65106, Bangkok, Thailand; National University of Singapore, Singapore, Singapore

**Keywords:** FATP2, fatty acid uptake, lipofermata, neuroblastoma, virus-lipid interaction, Zika

## Abstract

Zika virus (ZIKV) infection in adults is typically mild and self-limiting, but infection during pregnancy can cause fetal and neonatal microcephaly. No approved drugs or vaccines currently exist. ZIKV hijacks host lipid homeostasis to facilitate replication, and inhibitors of fatty acid synthesis decrease its production. However, cells obtain lipids from both *de novo* synthesis and uptake. Neurons, a primary target of ZIKV pathogenesis, have limited capacity for *de novo* lipid synthesis and rely more on lipid uptake. We previously demonstrated that lipid uptake inhibitors block influenza virus replication, yet their efficacy against ZIKV—particularly in neuronal cells—remains unknown. In the present study, we examined the anti-ZIKV and fatty acid uptake inhibitory activities of lipofermata, a fatty acid transporter protein isoform 2 (FATP2)‒specific inhibitor. Lipofermata effectively inhibited ZIKV production in immortalized hepatocyte (imHC) and SH-SY5Y (neuroblastoma) cell lines, with respective half-maximal inhibitory concentration (IC_50_) values of 1.41 and 1.11 μM with 50% cytotoxic concentration (CC_50_) of more than 20.0 μM. FATP2 mRNA and protein expression was significantly elevated in infected SH-SY5Y cells. ZIKV also significantly increased cellular phosphatidylcholine content. Lipofermata inhibited fatty acid uptake with an IC_50_ of 7.0 μM and significantly decreased phosphatidylcholine level. In addition, FATP2 knockdown by siRNA significantly decreased FATP2 mRNA and protein expression in SH-SY5Y and decreased ZIKV titer. These findings suggest that lipofermata disrupts ZIKV replication via fatty acid uptake inhibition. Furthermore, lipofermata at 2 µM markedly diminished caspase 3/7 and 8 activities, indicating that lipofermata mitigated ZIKV-induced extrinsic apoptosis in SH-SY5Y. Mitigating apoptosis may result in reduced neuropathogenesis. Consequently, FATP2 may serve as a promising target for anti-ZIKV therapies.

**IMPORTANCE:**

Zika virus can infect and cause severe damages to the brain. There is no effective antiviral drug for the treatment of Zika infection. Similar to many viruses, replication of Zika virus requires lipid. We show here that the virus could upregulate the fatty acid transporter FATP2 in neuroblastoma cells, which may increase the lipid supply in the infected cells, and that inhibiting the fatty acid transporter by lipofermata could suppress the viral replication and apoptosis. ZIKV production was also reduced by FATP2 knockdown. These suggest that FATP2 may be a promising target for the development of anti-Zika medication.

## INTRODUCTION

Zika virus (ZIKV) is a positive-sense, single-stranded RNA virus in the *Flaviviridae* family. It is a mosquito-borne virus (1). ZIKV was first isolated from a Rhesus monkey in Uganda in 1947 (2), and human infection was confirmed in Nigeria in 1954 (3). ZIKV infection in adults is normally mild and self-limiting. However, it became a serious disease because it is linked to microcephaly in fetuses and newborns (4) and Guillain‒Barré syndrome (5). Microcephaly can occur when pregnant women are infected, and a transplacental infection ensues (1). From the 1950s to 2019, ZIKV infection was reported in 87 countries, with the highest peak in the Americas in 2016 (6, 7). In Thailand, ZIKV has been reported since 2012 (8). In addition, the Bureau of Emerging Infectious Diseases, Department of Disease Control, Ministry of Public Health in Thailand reported that more than 1,600 cases were infected in 2016 and 2017 (9). Currently, there are no drugs or vaccines available against ZIKV infection.

ZIKV can take advantage of lipid droplets for replication. ZIKV production was decreased in the AUP1 (a lipid droplet-localized type III membrane protein) knockout in a HepG2 cell line (10). The ZIKV strain Argentina INEVH116141 was studied for its effect on lipid droplets in Huh-7, a human hepatocyte cell line. Investigators observed a significant reduction in lipid droplet volume per cell in ZIKV-infected cells, suggesting that ZIKV induces lipophagy in Huh-7 (11). The lipid profile reprogramming by ZIKV was studied in human placenta. ZIKV increased various phospholipids, including phosphatidylcholine (PC), phosphatidylethanolamine (PE), phosphatidylinositol (PI), and phosphatidylserine (PS), and also increased intracellular vesicle to support viral replication called replication complex (RC) (12). Flavivirus RC is generated from the rough endoplasmic reticulum (ER), where PC and PE are the major components (13–15). Therefore, PC and PE may enhance flavivirus and also ZIKV by augmenting the structural components of the replication complex structure.

Lipid-lowering drugs that inhibit ZIKV production include atorvastatin, cerivastatin, fluvastatin, lovastatin, mevastatin, and simvastatin (16). Metformin (17) and orlistat (18) also exert anti-ZIKV activity. These findings suggest that fatty acid synthesis is important for ZIKV production. However, the role of fatty acid uptake in ZIKV replication has never been examined. Our previous findings revealed that lipid uptake inhibitors can suppress influenza virus replication (19). Thus, it is plausible that a similar strategy may be effective against ZIKV.

Cells can acquire fatty acids through multiple pathways, including passive diffusion (flip-flop) (20), fatty acid translocase (CD36) (21), fatty acid transporter proteins (FATP) (22, 23), and caveolin-1 (24). Different cell types may rely on distinct transporters. Among the FATP isoforms, FATP2 has emerged as a promising target because FATP2 knockdown (via short hairpin RNA) reduces fatty acid uptake (23). Moreover, suppressing FATP2 attenuates fatty acid transport and protects mice from diet-induced nonalcoholic fatty liver disease (25). A specific FATP2 inhibitor, lipofermata, is currently under development as a potential anticancer agent (26, 27). Although FATP1 and FATP4 are the main fatty acid transporters in the brain, no inhibitors are available for these isoforms. Notably, FATP2 is also expressed in the brain (28).

Previous studies have shown that ZIKV infection affects fetuses and can cause severe neurological complications. ZIKV can induce activities of caspases 3/7, 8, and 9 in neuronal progenitor cells. Neuropathogenesis is believed to originate from apoptosis and also lead to microcephaly in newborns (29). Human hepatic cells and human neuronal progenitor cells are prime targets for ZIKV infection (30). A stem cell-derived cell line, an immortalized hepatocyte-like cell line (imHC), was used in this study. It was developed from the human mesenchymal stem cells (hMSCs) to a hepatocyte-like phenotype (31). It is a suitable *in vitro* model of dengue virus infection, including pathogenesis and anti-viral testing with FATP2 expression (31–33). The human neuroblastoma cell line SH-SY5Y has also been employed as a neuron-based model for ZIKV infection. ZIKV-infected SH-SY5Y cells display slight changes in viability, cytotoxicity, and morphology, although viral particles have been detected in the nucleoplasmic compartment (34). Several studies have used SH-SY5Y cells to investigate ZIKV infection (35–37). Moreover, FATP2 is expressed in SH-SY5Y cells (38).

In the current study, we investigated the anti-ZIKV activity of lipofermata, an FATP2 inhibitor, in an immortalized human hepatic cell line and in SH-SY5Y cells. We also analyzed the degree of lipid uptake inhibition and cellular lipid decreasing by lipofermata in SH-SY5Y cells. Reduction of ZIKV-induced apoptosis by lipofermata was determined. We also examined FATP2 mRNA and protein expression in SH-SY5Y cells during ZIKV infection. Moreover, FATP2 function for ZIKV production was investigated by silencing FATP2.

## MATERIALS AND METHODS

### Cells and viruses

Immortalized hepatocyte (imHC) (31) and neuroblastoma (SH-SY5Y, CRL-2266) cell lines were maintained in Dulbecco’s Modified Eagle Medium/Nutrient Mixture F12 1:1 (DMEM/F12; Cytiva) supplemented with 15% inactivated fetal bovine serum (FBS; Gibco). The *Aedes albopictus* mosquito cell line (C6/36, CRL-1660, ATCC) and the African green monkey kidney epithelial cell line (Vero, CCL-81, ATCC) were cultured in Leibovitz’s L-15 Medium (L15; Gibco) and Minimum Essential Medium (MEM; Gibco), respectively. All cell lines were cultured in antibiotic-free medium.

ZIKV clinical isolate MU-DMSC-4/2017 (39) was obtained from leftover serum samples after diagnostic testing for ZIKV infection between 2016 and 2018. These samples were provided by the Regional Medical Sciences Centers and the National Institute of Health, Department of Medical Sciences, Ministry of Public Health. ZIKV was isolated in *Toxorhynchites splendens* mosquitoes and identified by immunofluorescence assay and real-time RT-PCR targeting the NS2B region of the viral genome. The virus was propagated in C6/36 cells, and the resultant supernatant was stored at −80°C without refreezing.

### Chemicals

Lipofermata (CAY-25869, Cayman Chemical Company) was dissolved in DMSO (D2650, Sigma-Aldrich). Camptothecin (C1495, Tokyo Chemical Industry Co., Ltd.) was also dissolved in DMSO and used as a positive control of apoptosis experiment (40). The resulting solution was aliquoted and stored at −80°C, avoiding multiple freeze‒thaw cycles.

### Cell viability assay

PrestoBlue cell viability reagent (A13261, Invitrogen) was used. SH-SY5Y or imHC cells were seeded in 96-well plates and incubated at 37 °C with 5% CO_2_ for 18‒24 h to reach approximately 90% confluence. After a 48-h incubation with lipofermata, the supernatant was removed. Next, 1× PrestoBlue in phenol red‒free DMEM/F12 was added, and cells were incubated at 37 °C with 5% CO_2_ for 30 min. Fluorescence intensity (excitation at 560 nm, emission at 590 nm) was measured using a Synergy H1 microplate reader. The 50% cytotoxic concentration (CC_50_) was calculated by comparing to 2% FBS 1% DMSO DMEM/F12-treated group (control). Experiments were performed in triplicate.

### Evaluation of anti-ZIKV activity of lipofermata

To assess the anti-ZIKV effect of lipofermata in imHC and SH-SY5Y cells, 96-well plates designated for SH-SY5Y were first coated with poly-D-lysine for 30 min at room temperature. After discarding the poly-D-lysine solution, plates were rinsed with sterile Milli-Q water and air-dried. Both SH-SY5Y and imHC cells were seeded and incubated at 37 °C with 5% CO_2_ for 18‒24 h. The supernatant was then removed, and cells were infected with 50 µL of ZIKV at a multiplicity of infection (MOI) of 1 for imHC or 5 for SH-SY5Y. Infection proceeded at 37 °C with 5% CO_2_ for 2 h, after which the virus supernatant was discarded. Various concentrations of lipofermata in DMEM/F12 containing 2% FBS and 1% DMSO were then added, and cells were incubated at 37 °C with 5% CO_2_ for 48 h. Experiments were performed in triplicate. The half-maximal inhibitory concentration (IC_50_) was determined by comparing to 2% FBS 1% DMSO DMEM/F12 treated group (control).

### Virus titer determination by focus immunoassay

The focus immunoassay was adapted from a previous report (41). Briefly, 50 µL of diluted virus was added to Vero cells and incubated at 37 °C with 5% CO_2_ for 2 h. Then, 125 μL of overlay medium (1.5% [w/v] carboxymethyl cellulose [C4888, Sigma-Aldrich] in 2% FBS MEM) was added, and cells were incubated for 2 days. Afterward, cells were washed with 1× phosphate-buffered saline (PBS), fixed with 3.7% (v/v) formaldehyde (Sigma‒Aldrich) for 15 min, and permeabilized with 2% (v/v) Triton X-100 surfactant (OmniPur) for 10 min.

Next, cells were incubated with anti-E monoclonal antibody (4G2) at 37 °C for 1 h. After washing, cells were incubated with goat anti-mouse IgG (H + L)-AP (ab97020; Abcam) at 37 °C for 1 h. The cells were then washed and incubated in the dark for 15 min with a substrate solution of nitro-blue tetrazolium and 5-bromo-4-chloro-3′-indolyphosphate (ab146262, ab146226; Abcam). Once a violet color developed, the cells were washed with tap water. Foci were photographed using an ImmunoSpot analyzer (S6 Ultimate, Cellular Technology Limited, Cleveland, OH, USA), and the virus titer (FFU/mL) was calculated.

### Reverse transcriptase real-time PCR for determination of FATP2 mRNA expression

To assess FATP2 mRNA expression in SH-SY5Y cells, 24-well plates were first coated with poly-D-lysine for 30 min at room temperature. After discarding the solution, the plates were rinsed with sterile Milli-Q water and air-dried. SH-SY5Y cells were then seeded and incubated at 37 °C with 5% CO_2_ for 18‒24 h. Once the medium was removed, cells were infected with ZIKV at an MOI of 5 and incubated at 37 °C with 5% CO_2_ for 2 h. After discarding the virus supernatant, lipofermata (1 μM) was added, and cells were further incubated at 37 °C with 5% CO_2_. At 24 and 48 h post-infection (p.i.), cells were collected, and total RNA was extracted by the Trizol method.

For RNA extraction, Trizol reagent was added directly to the cells, mixed by pipetting, and incubated at 4°C for 5 min. Chloroform (0.2 mL per mL of Trizol) was then added and briefly vortexed, and the mixture was incubated at 4°C for 5 min. After centrifugation at 12,000 × *g* for 20 min at 4°C, the aqueous phase was transferred to a new tube, and isopropanol (0.5 mL per mL of Trizol) was added. Following brief vortexing and a 10-min incubation at 4°C, samples were centrifuged at 12,000 × *g* for 10 min to precipitate RNA. The supernatant was discarded, and the pellet was washed with 1 mL of 75% ethanol, mixed by vortexing, and centrifuged at 7,500 × *g* for 5 min at 4°C. After removing the supernatant, the pellet was vacuum-dried and dissolved in sterile Milli-Q water. The RNA concentration was measured by NanoDrop.

FATP2 mRNA levels were quantified using the SensiFAST SYBR No-ROX One-Step Kit (BIO-72001, Meridian Bioscience) with the previously described primers (42). Briefly, the forward primer was 5′-TCTTGGATGACACAGCAAAAATGT-3′, and the reverse primer was 5′-TCAGAGTTTCAGGGTTTTAGCACTT-3′. GAPDH (forward primer: 5′-CAACTACATGGTTTACATGTTC-3′; reverse primer: 5′-GCCAGTGGACTCCACGAC-3′) served as an internal control (43). RNA was incubated at 60°C for 10 min before adding the template. All experiments were performed in triplicate.

### Western blotting for measurement of FATP2 protein expression

The SH-SY5Y was seeded into a six-well plate and incubated at 37°C with 5% CO_2_ for 18–24 h to 90% confluent. The SH-SY5Y was infected with ZIKV at an MOI of 5. After 48 h p.i., protein was extracted by pre-cooled radioimmunoprecipitation assay buffer (RB4475, Bio Basic). Ten micrograms of protein was separated by using a NuPAGE 4–12% Bis-Tris Gel (NP0322BOX; Invitrogen) with electrophoresis (110 V for 2 h). Protein bands were transferred onto a nitrocellulose membrane (GE Healthcare) by non-electroblotting for 3 days at RT and washed with 0.1% (v/v) Tween20 (97063-872, AMRESCO) in PBS (0.1% PBST) with rocking for 5 min (three times). Membrane was blocked with 3% (w/v) skim milk in 0.1% PBST (antibody diluent) and incubated at RT for 1 h with rocking. Then, the membrane was incubated with rabbit anti-FATP2 polyclonal antibody (1:1,000, ab83763, Abcam) at 4°C overnight. Membrane was washed and incubated with goat anti-rabbit antibody conjugated HRP (1:2,000, P0448, Dako) at RT for 2 h. The ECL western blotting substrate (1705061, Bio-Rad) was added to develop chemiluminescent signal and incubated in the dark at RT for 3 min. Membrane was photographed by iBright CL1500 Imaging System (Invitrogen). Protein band intensity was analyzed by iBright analysis software (Invitrogen). To detect GAPDH protein, the same membrane was washed and incubated with mouse anti-GAPDH monoclonal antibody (1:10,000, sc-32233, Santa Cruz Biotechnology) at 4°C overnight. Then, the membrane was incubated with rabbit anti-mouse antibody conjugated HRP (1:5,000, P0260, Dako) at RT for 2 h. Chemiluminescent signal was detected as described earlier.

### Evaluation of long-chain fatty acid uptake inhibition

To assess the effect of lipofermata on fatty acid uptake in SH-SY5Y cells, we used a method adapted from a previous report (44). A black-walled, clear-bottom 96-well plate was first coated with poly-D-lysine for 30 min at room temperature. After discarding the solution, the plate was rinsed once with sterile Milli-Q water and air-dried. SH-SY5Y cells were then seeded and incubated at 37 °C with 5% CO_2_ for 18‒24 h.

Next, the medium was removed, and cells were incubated in phenol red-free DMEM/F12 containing 1% DMSO, with or without lipofermata, at 37 °C with 5% CO_2_ for 1 h. Subsequently, we added 100 µL of a solution containing 4,4-difluoro-5,7-dimethyl-4-bora-3a,4a-diaza-s-indacene-3-hexadecanoic acid (BODIPY FL C16; D3821, Invitrogen) conjugated with fatty acid-free bovine serum albumin (A8806, Sigma‒Aldrich) at a 1:2.5 ratio, along with lipofermata and 0.125% (w/v) Trypan blue (Gibco) in phenol red-free DMEM/F12. Cells were incubated at 37 °C with 5% CO_2_ for 30 min. Fluorescence intensity (excitation 485 nm; emission 528 nm) was then measured with a Synergy H1 microplate reader (BioTek). Experiments were performed in triplicate, and the percentage of lipid uptake inhibition was calculated by comparing to 2% FBS 1% DMSO DMEM/F12-treated group (control).

### Cellular lipid quantitation by thin-layer chromatography 

To evaluate lipofermata activity on cellular lipid, the SH-SY5Y was seeded into a six-well plate and incubated at 37°C with 5% CO_2_ for 18–24 h to 90% confluent. The SH-SY5Y was adsorbed with ZIKV (MOI of 5) at 37°C with 5% CO_2_ for 2 h. Then, the cell was incubated with 2 µM of lipofermata in 2% FBS 1% DMSO DMEM/F12 for 48 h. Mock and virus control were incubated with 2% FBS 1% DMSO DMEM/F12. Supernatant was collected to quantify ZIKV titer, and cells were collected to extract lipid by using Bligh and Dyer’s method (45). Cells were collected by cell scraper and washed once with 1× PBS. Cells were centrifuged at 1,500 rpm for 5 min. After discarding the supernatant, the cell pellet was resuspended with 400 uL of 1× PBS and transferred to a capped-glass tube. MilliQ water 400 µL was added to rinse microcentrifuge tube and transfer to same capped-glass tube. Methanol and chloroform (2 and 1 mL, respectively) were added to the reaction tube. After briefly vortexing, they were incubated at RT for 30 min. Chloroform and MilliQ water in each 1 mL were added, briefly vortexed, and centrifuged at 2,000 × *g* 25°C for 3 min. The lower chloroform phase was transferred to a screw-capped glass bottle, and the extracted lipid was dried with nitrogen gas. Dried lipid was resuspended with 20 µL of chloroform. To activate F256 HPTLC silica plate (5631-5, Merck, Darmstadt, Germany), silica plate was soaked by capillary with methanol/chloroform (30/30 mL) in thin-layer chromatography (TLC) chamber and hot air-dried. A lipid solution was applied to a silica plate and air-dried. Lipid standards in each 25 µg that included phosphatidylethanolamine (PE), phosphatidylcholine (PC), and galactosylceramide (GalCer) were co-chromatographed with the lipid samples. Lipid was separated by using stationary phases (chloroform 65 mL:methanol 25 mL:MilliQ water 10 mL) (46). Silica plate was air-dried and stained with Coomassie brilliant blue dye (0.03% brilliant blue G [27815, Sigma]:30% methanol:100 mM NaCl in MilliQ water) for 15 min at RT. Then, silica plate was de-stained with de-stain buffer (30% methanol: 100 mM NaCl in MilliQ water) for 15 min at RT and air-dried. Silica plate was photographed by iBright CL1500 Imaging System (Invitrogen). Lipid band intensity was analyzed by iBright analysis software (Invitrogen). Experiment was performed in triplicate.

### Measurement of lipofermata-reduced apoptosis in ZIKV-infected SH-SY5Y

Apoptosis signals were quantitated by using Caspase 3/7, 8, and 9 Multiplex Activity Assay Kit (ab219915, Abcam). The kit detects caspase 3/7, 8, and 9 activities by using DEVD-ProRe, IETD-R110, and LEHD-AMC as fluorogenic indicators, respectively. The SH-SY5Y cell was seeded into pre-coated poly-D-lysine black-wall, clear flat-bottom 96-well plate and incubated at 37 °C with 5% CO_2_ for 18‒24 h. Cells were adsorbed with ZIKV at an MOI of 5 at 37 °C with 5% CO_2_ for 2 h. Lipofermata at 1 and 2 µM in 2% FBS 1% DMSO DMEM/F12 were added and incubated at 37 °C with 5% CO_2_ for 48 h. Mock and virus control was incubated with 2% FBS 1% DMSO DMEM/F12. Supernatant was collected to quantify ZIKV titer. Caspase 3/7, 8, and 9 substrates in assay buffer 100 µL were added to cell and incubated in dark for 1 h at RT. Plate was measured for fluorescence intensity with excitation/emission at 535/620, 490/525, and 370/450 nm for caspase 3/7, 8, and 9, respectively. Camptothecin 5 µM was used as a positive control. Experiments were performed in triplicate.

### FATP2 protein silencing by using siRNA for decreasing ZIKV production

To evaluate FATP2 function for ZIKV production, FATP2 protein was silenced by siRNA. The SH-SY5Y cell was seeded into a 12-well plate and incubated at 37 °C with 5% CO_2_ for 18 h with 50% confluence. Cell was transected with FATP2 siRNA (ON-TARGETplus Human SLC27A2 [11001] siRNA—SMARTpool, L-007498-00-0005, Horizon) with 50 nM and 5 µL of Lipofectamine 2000 (11668027, Invitrogen) in 500 µL of Opti-MEM I medium (31985070, Gibco). Then, the plate was incubated at 37 °C with 5% CO_2_ for 24 h. Medium was replaced with 15% FBS DMEM/F12 and incubated for 24 h. Transfected cells were collected for real-time RT PCR to determine FATP2 mRNA expression. After incubating for 4 days, the transfected cell was collected to quantitate FATP2 protein expression by western blot. Another transfected cell was adsorbed with ZIKV at an MOI of 5 at 37 °C with 5% CO_2_ for 2 h. Medium was replaced with 2% FBS DMEM/F12 and incubated at 37 °C with 5% CO_2_ for 48 h. Supernatant was collected to quantitate ZIKV titers. ZIKV titers were compared between FATP2 siRNA and negative siRNA (ON-TARGETplus Non-Targeting Pool, D-001810-10-05, Horizon). The experiment was performed in triplicate.

### Statistical analysis

The GraphPad Prism software version 10.5.0 was used to analyze the statistics. Data of triplicate experiments are presented as the mean ± SD (error bar). The difference between groups was compared using Student’s *t*-test. A significance threshold of *P* < 0.05 was established. The CC_50_ and IC_50_ were analyzed by using the method “inhibitor vs. normalized response−variable slope”.

## RESULTS

### Anti-ZIKV activity of lipofermata in imHC and SH-SY5Y cell lines

**Fig 1:**
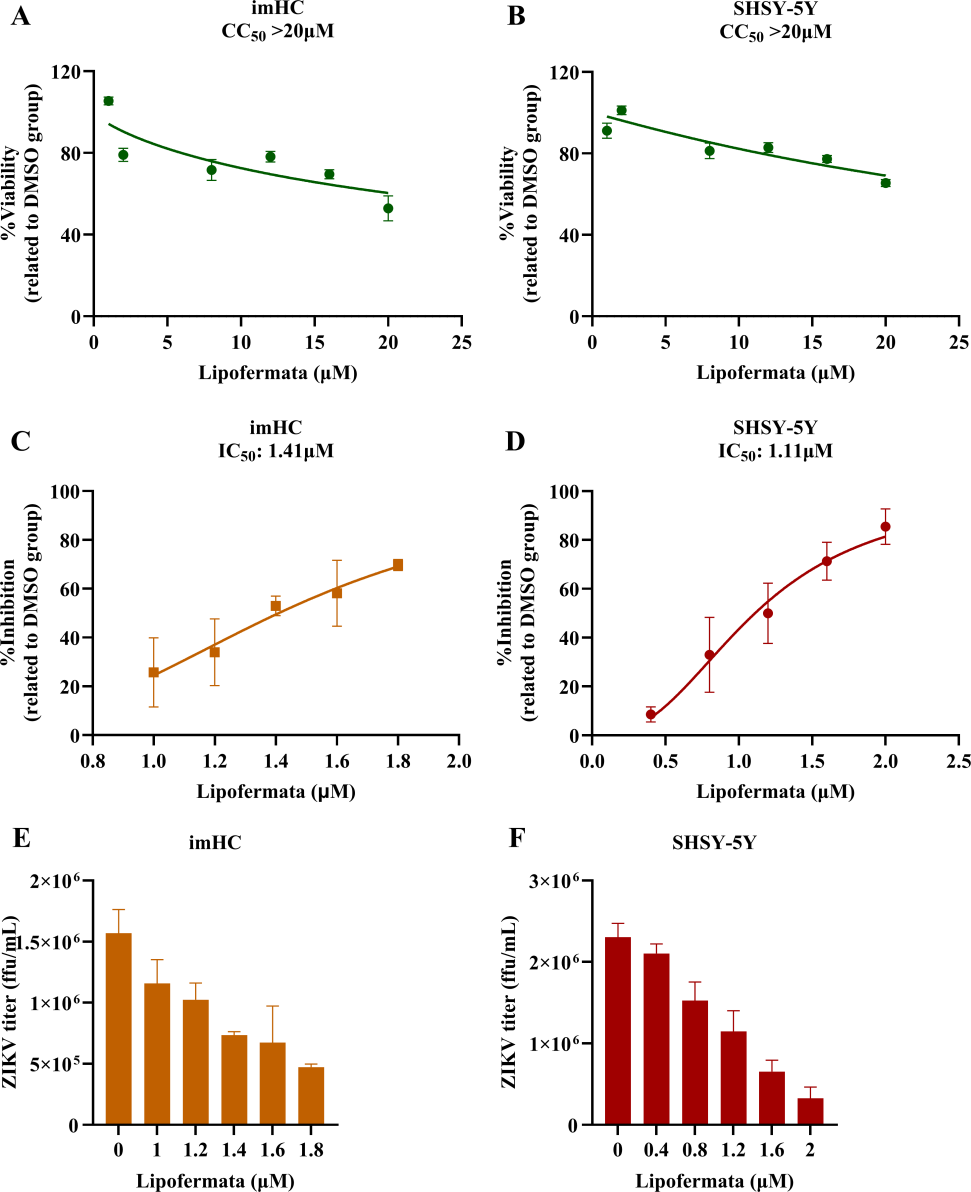
Lipofermata exhibits concentration-dependent cytotoxicity and anti-ZIKV activity in imHC and SH-SY5Y cells. The line graphs show cell viability at 48 h post-infection in imHC (**A**) and SH-SY5Y (**B**) cells. ZIKV inhibition activity of lipofermata in imHC (**C**) and SH-SY5Y (**D**) is shown. The *X* axis represents the concentration of lipofermata (µM). The *Y* axis represents viability (%) and inhibition (%) related to the DMSO group. Viral inhibition in imHC (**E**) and SH-SY5Y (**F**) was quantified by focus immunoassay. The *X* axis represents the concentration of lipofermata (µM). The *Y* axis represents ZIKV titer (ffu/mL) related to the DMSO group. Values are the means with standard deviations (error bar) from three independent experiments.

We tested the anti-ZIKA activity of lipofermata in two major ZIKA target cell types, hepatocyte and neuron. Various concentrations of lipofermata were applied to imHC and SH-SY5Y cells. The supernatant was collected to measure viral titer, and cell viability was assessed in parallel. The CC_50_ values were more than 20 µM in imHC (Fig. 1A) and SH-SY5Y (Fig. 1B). The IC_50_ values were 1.41 and 1.11 μM in imHC (Fig. 1C) and SH-SY5Y (Fig. 1D), respectively. ZIKV titer decreased following lipofermata treatment in a dose-dependent manner with imHC (Fig. 1E) and SHSY-5Y (Fig. 1F). These findings indicate that lipofermata inhibits ZIKV in these two cell lines representing the major target cell types.

### FATP2 mRNA expression during ZIKV infection in the SH-SY5Y cell line

**Fig 2:**
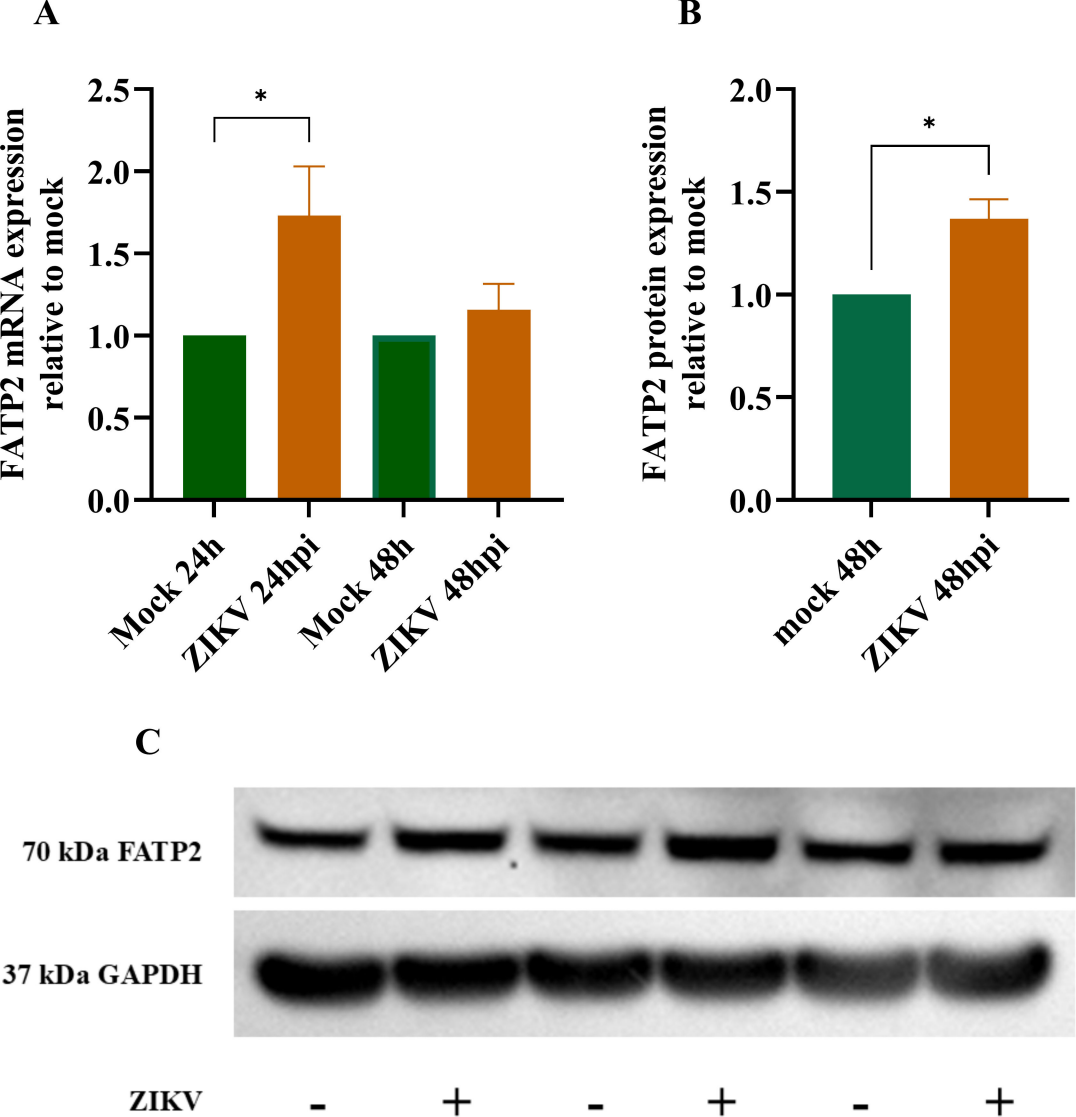
ZIKV infection up-regulates FATP2 mRNA and protein in SH-SY5Y cells. The bar graphs represent FATP2 mRNA (**A**) and protein (**B**) levels of the ZIKV-infected SH-SY5Y cell line. The *X* axis represents the ZIKV hours post-infection. The *Y* axis represents the relative expression to mock with normalized to the GAPDH. The picture represents FATP2 level in the western blot membrane of ZIKV infection compared to mock (**C**). Data are the means with standard deviations (error bar) of three independent experiments. Student’s *t*-test was used to compare a difference between groups. Statistical significance: *P < 0.05, h: hour, hpi: hours post-infection; kDa: kilodalton.

To investigate FATP2 mRNA expression in SH-SY5Y cells during ZIKV infection, cells were infected at an MOI of 5 for 2 h. After removing the supernatant, culture medium containing 1% DMSO and 2% FBS in DMEM/F12 was added. Total RNA was extracted at 24 and 48 h p.i. Real-time reverse transcription PCR was then performed to quantify FATP2 mRNA levels, which were normalized against GAPDH. The results showed that ZIKV infection significantly increased FATP2 expression in SH-SY5Y cells at 24 h p.i. (*P* < 0.05; Fig. 2A). Thus, ZIKV could at least transiently increase FATP2 expression at an early time point. This up-regulation may help increase fatty acid uptake to provide the virus with more abundant lipid supply for energy metabolism and membrane expansion, which are needed for active viral replication.

### FATP2 protein expression during ZIKV infection in the SH-SY5Y cell line

FATP2 was detectable in various band sizes as described by the manufacturer (ab83763, Abcam). Nonetheless, the manufacturer disclaimed that the expected band is approximately 70 kDa. Therefore, the 70 kDa band was selected to analyze in the experiment. At 48 h p.i., FATP2 expression was significantly increased by ZIKV infection (*P* < 0.05) (Fig. 2B and C). To confirm the infection, ZIKV titer was 1.36 ± 0.47 × 10^6^ ffu/mL at 48 h p.i. Thus, FATP2 expression was induced by ZIKV infection in SH-SY5Y cells.

### Lipofermata inhibited long-chain fatty acid uptake in the SH-SY5Y cell line

**Fig 3:**
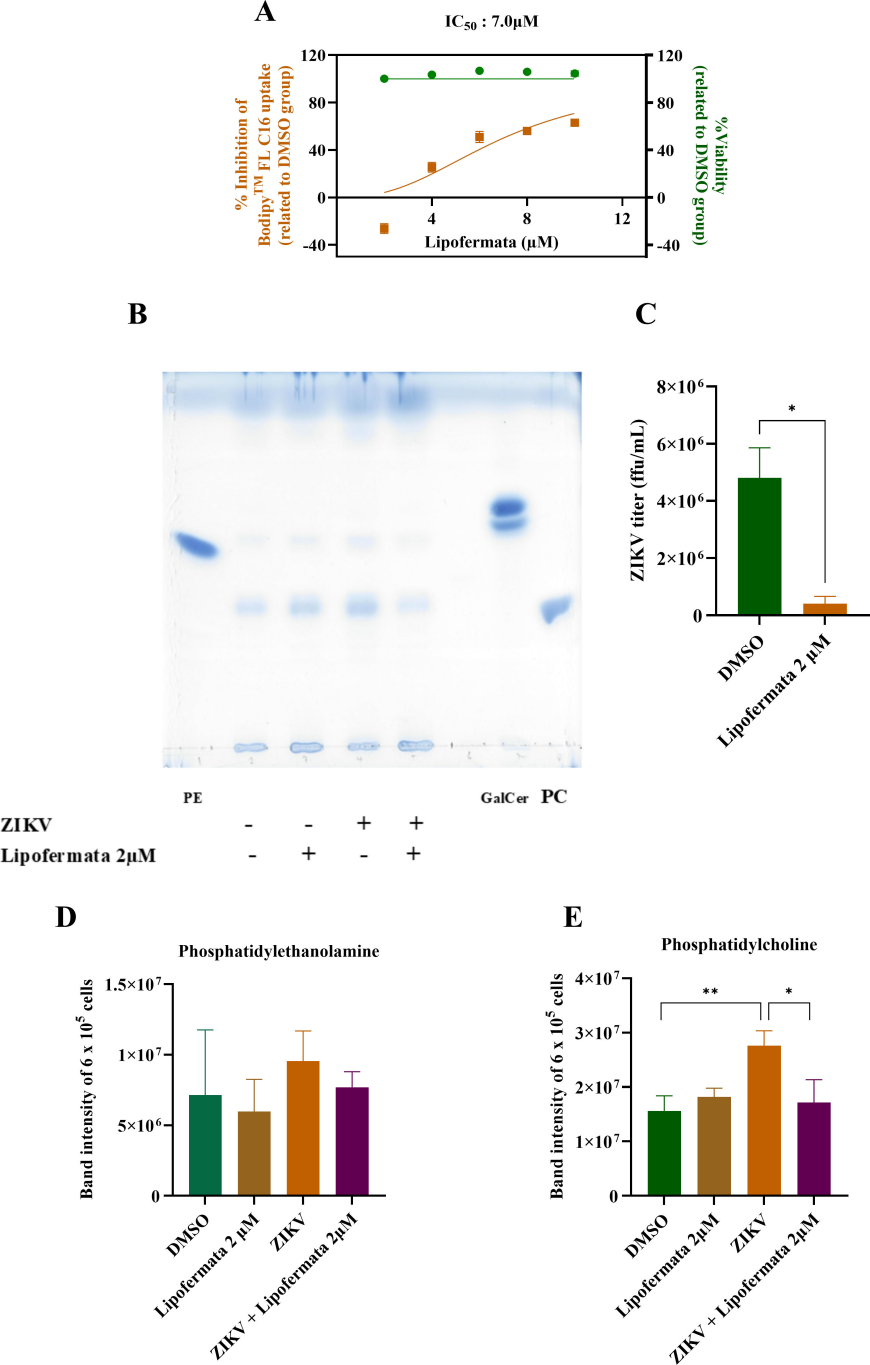
Lipofermata inhibits BODIPY-labeled palmitate uptake and decreases phosphatidylcholine in SH-SY5Y cells. SH-SY5Y cells were lipid-starved for 1 h in serum-free DMEM/F12 containing lipofermata, and then incubated with BODIPY FL C16 and the same lipofermata concentrations for an additional hour. Fluorescence (excitation 485 nm, emission 528 nm) and cell viability (PrestoBlue) were recorded. The graph shows the percentage viability (green) and the percentage inhibition of fatty-acid uptake (orange) (**A**). The *X* axis represents the concentration of lipofermata (µM). The *Y* axis represents viability (%) and inhibition (%) of BODIPY FL C16 uptake related to the DMSO group. Photo depicts lipid separation by using the TLC method in treatment of lipofermata with ZIKV-infected SH-SY5Y (**B**). The bar graph represents ZIKV titer from TLC experiment (**C**). The *X* axis represents the concentration of lipofermata (µM). The *Y* axis represents ZIKV titer (ffu/mL). The bar graph represents band intensity of phosphatidylethanolamine (**D**) and phosphatidylcholine (**E**) that measure from TLC. The *X* axis represents the treatment condition. The *Y* axis represents lipid band intensity. Data are the means with standard deviations (error bar) from three independent experiments. Student’s *t*-test was used to compare a difference between groups. Statistical significance: *P < 0.05; **P < 0.005, PE: phosphatidylethanolamine; PC: phosphatidylcholine; GalCer: galactosylceramide.

To confirm that lipofermata inhibits the uptake of long-chain fatty acids in SH-SY5Y cells, we treated cells with lipofermata, and then incubated them with BODIPY FL C16, a fluorescently labeled palmitic acid. Fluorescence intensity was subsequently measured. Lipofermata reduced BODIPY FL C16 uptake with an IC_50_ of 7.0 μM without detectable cytotoxicity in SH-SY5Y cells. The lack of cytotoxicity at this concentration comparable to the CC_50_ in the antiviral experiment was because of the much shorter exposure time required in this experiment. The level of the lipid uptake inhibition was comparable to previous reports on lipofermata (25). Hence, lipofermata effectively inhibited long-chain fatty acid uptake in the SH-SY5Y cell line (Fig. 3A).

### Lipofermata decreased phosphatidylcholine in SH-SY5Y

To clarify lipid uptake inhibition activity of lipofermata at the ending of incubation period, cells were collected to extract cellular lipid at 48 h p.i.. ZIKV titer was significantly decreased by lipofermata at 2 µM from 4.80 ± 1.05 × 10^6^ ffu/mL to 4.13 ± 2.45 × 10^5^ ffu/mL (Fig. 3C). After lipid was separated by the TLC method, at least four dominant bands appeared (Fig. 3B). However, two band positions were equivalent to PC and PE control. These PE (Fig. 3D) and PC (Fig. 3E) intensities were increased by ZIKV infection and decreased by lipofermata. However, only PC was significantly increased by ZIKV (*P* < 0.005) and decreased by lipofermata at 2 µM (*P* < 0.05). Lipofermata alone did not significantly increase PC intensities. Thus, lipofermata reduced ZIKV-induced PC in SH-SY5Y.

### Lipofermata reduced caspase 3/7 and 8 activities in SH-SY5Y cell line

**Fig 4:**
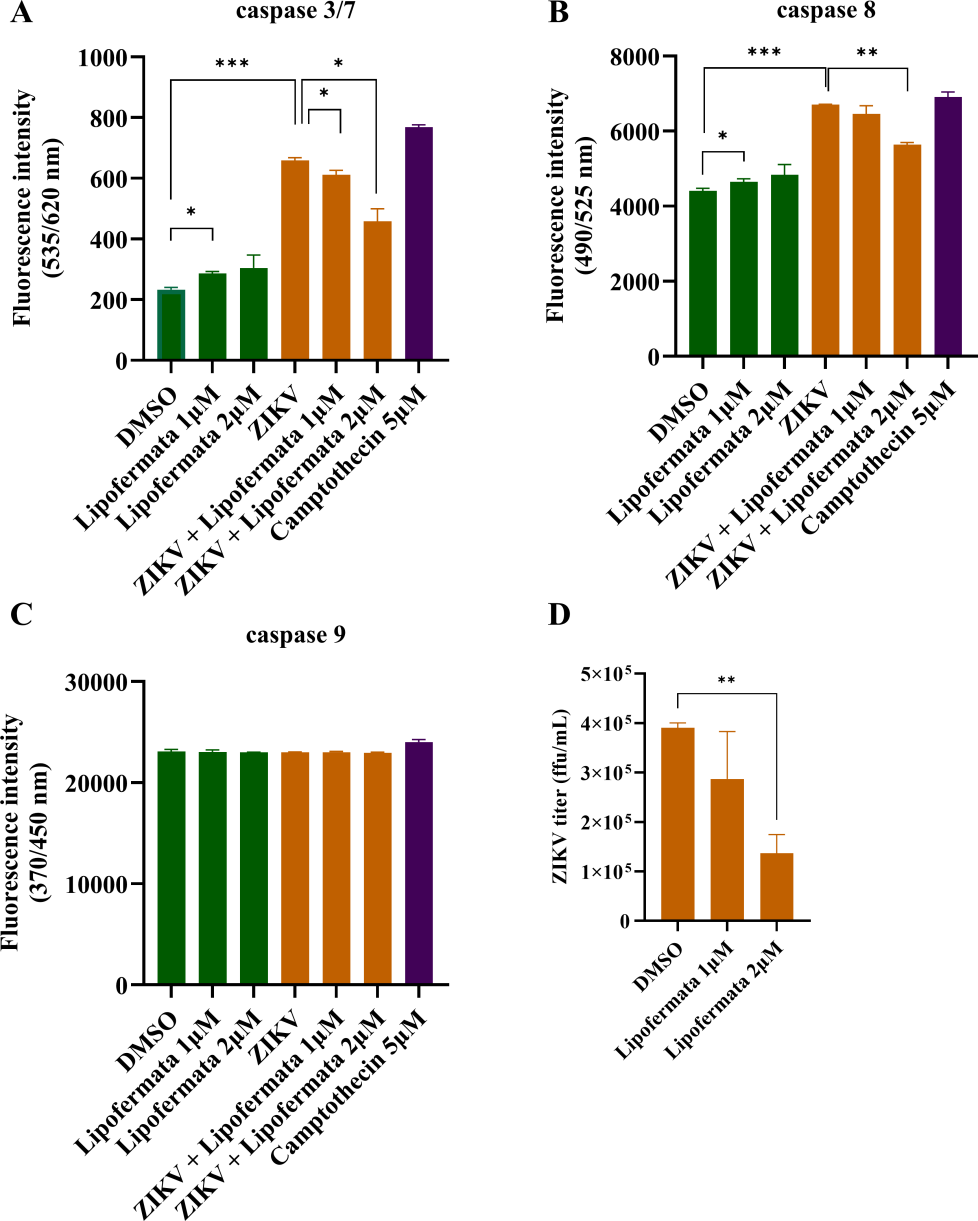
Lipofermata decreased caspase 3/7 and 8 activities in ZIKV-infected SH-SY5Y cells. ZIKV-infected SH-SY5Y cells were treated with lipofermata at 1 and 2 µM for 48 h p.i. Then, caspase 3/7, 8, and 9 activities were measured by fluorometric method. The bar graphs represent the fluorescence intensity of caspase 3/7 (**A**), caspase 8 (**B**), and caspase 9 (**C**). The *X* axis represents the condition that was treated to the cell. The *Y* axis represents fluorescence intensity. The bar graph represents ZIKV titer from caspase activity experiment (**D**). The *X* axis represents the concentration of lipofermata (µM). The *Y* axis represents ZIKV titer (ffu/mL). Data are the means with standard deviations (error bar) from three independent experiments. Student’s *t*-test was used to compare a difference between groups. Statistical significance: *P < 0.05; **P < 0.005; ***P < 0.0005.

To evaluate lipofermata activity for decreasing ZIKV-induced apoptosis, caspase 3/7, 8, and 9 (Fig. 4C) activities were measured by fluorometric method. Caspase 3/7 (Fig. 4A) and 8 (Fig. 4B) were significantly increased by ZIKV infection (*P* < 0.0005). Interestingly, lipofermata at 1 and 2 µM could significantly decrease caspase 3/7 activities (*P* < 0.05) in ZIKV-infected SHSY5Y. Lipofermata at 2 µM significantly decreased caspase eight activity (*P* < 0.005). ZIKV titer was significantly decreased in a dose-dependent manner but significantly decreased at 2 µM from 3.90 ± 0.10 × 10^5^ ffu/mL to 1.10 ± 0.38 × 10^5^ ffu/mL (Fig. 4D). Lipofermata alone significantly increased caspase 3/7 and 8 (*P* < 0.05) but less than ZIKV infected alone. However, caspase nine activity was not significantly altered by ZIKV infection and/or lipofermata treatment. Camptothecin at 5 µM (positive control) elevated caspase 3/7, 8, and 9 activities in SH-SY5Y. Thus, lipofermata was able to decrease ZIKV-induced extrinsic apoptosis via decreasing of caspase 3/7 and 8 activities.

### FATP2 silencing by siRNA impaired ZIKV production in SH-SY5Y

**Fig 5:**
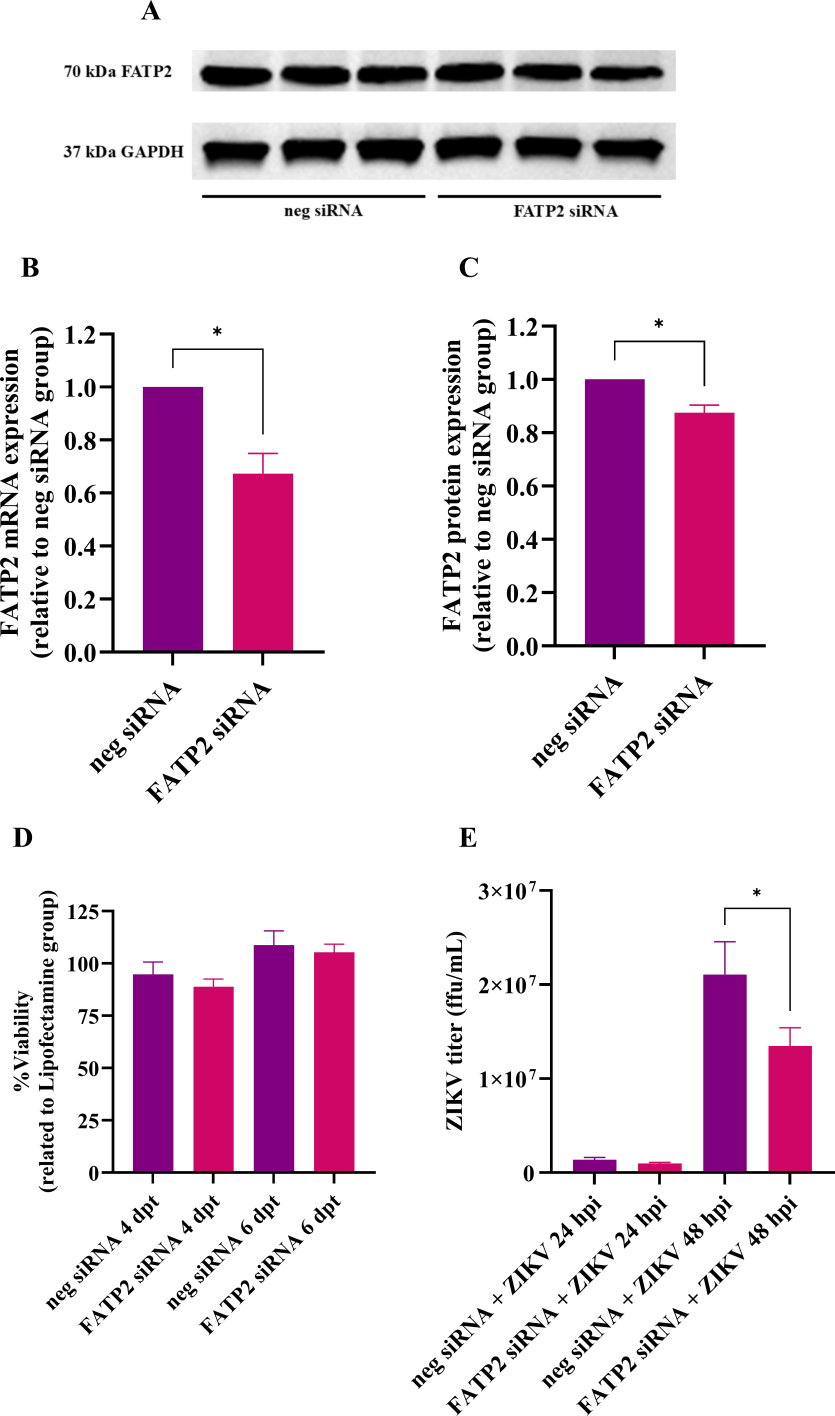
FATP2 silencing by using siRNA decreases ZIKV titer in SH-SY5Y cell line. FATP2 silencing was conducted in SH-SY5Y using siRNA, then infected with ZIKV. Picture depicts western blot membrane of FATP2 expression (**A**). The bar graphs represent FATP2 mRNA (**B**) and protein (**C**) expression in FATP2 knockdown experiment. The *X* axis represents the condition of siRNA. The *Y* axis represents FATP2 expression level (related to neg siRNA group). The bar graphs represent cell viability (%) in each condition (**D**). The *X* axis represents the condition of siRNA. The *Y* axis represents viability (%). The bar graph represents ZIKV titer in each condition (**E**). The *X* axis represents the condition of siRNA in 24 and 48 h p.i. The *Y* axis represents ZIKV titer (ffu/mL). Data are the means with standard deviations (error bar) from three independent experiments. Student’s *t*-test was used to compare a difference between groups. Statistical significance: * *P* < 0.05, dpt: days post transfection, hpi: hours post-infection.

To silence FATP2, FATP2 siRNA was transfected to SH-SY5Y cell line. After 48 h post-transfection, FATP2 mRNA was significantly decreased compared to negative siRNA control (Fig. 5B) (*P* < 0.05). After 96 h post-transfection, FATP2 protein expression was significantly decreased compared to negative siRNA control (Fig. 5A and C) (*P* < 0.05). Cell viability was not significantly decreased compared to the transfectant control group (Fig. 5D). After ZIKV infection for 24 and 48 h p.i., ZIKV titer was significantly decreased compared with neg siRNA control group. ZIKV titer was decreased from 1.35 ± 0.27 × 10^6^ to 9.60 ± 1.22 × 10^5^ ffu/mL at 24 h p.i. and significantly decreased from 2.10 ± 0.35 × 10^7^ to 1.34 ± 0.19 × 10^7^ ffu/mL (*P* < 0.05) (Fig. 5E). Therefore, FATP2 is essential for ZIKV production in SH-SY5Y.

## DISCUSSION

ZIKV infection is associated with microcephaly in newborns, particularly when exposure occurs at later stages of gestation. In human fetal brain tissues, the subventricular zone is a critical site of ZIKV infection. The main targets are intermediate progenitor cells, which are more vulnerable than post-mitotic neurons (47).

Lipids are vital for virus replication because they supply energy and facilitate the formation of replication organelles and viral envelopes. Disrupting lipid metabolism is, therefore a promising antiviral approach. While hepatocytes and adipocytes constitute major sites of fatty acid synthesis, other cell types predominantly rely on fatty acid uptake for their lipid supply. This uptake can proceed by simple diffusion or through active transporters. FATP2 has been a prime target for inhibitor development because it mediates lipid transport in the intestine, liver, and kidney. Inhibiting FATP2 can reduce lipid absorption and improve metabolic profiles, thus ameliorating fatty liver (23). Although primarily expressed in the intestine, liver, and kidney, FATP2 is also detected in other tissues, including the brain (28), and it is highly expressed in the placenta (48), another key target of ZIKV infection and pathogenesis. Therefore, FATP2 inhibitors offer an attractive therapeutic option with potential for *in vivo* efficacy.

Although glucose is the primary energy source for the brain, up to 20% of total energy can be derived from fatty acid oxidation in mitochondria (49). Glial cells assist and regulate neuronal lipid metabolism (50), and lipids are also essential for neuronal function because they serve as structural components, such as myelin. A peroxisome proliferator-activated receptor (PPAR) response element in the *Slc27a1* promoter enables binding and enhances FATP1 expression (51). In addition, PPARα and PPARγ regulate the expression of FATP2 and FATP4 (52–54). These nuclear receptors are activated by fatty acids and other lipid ligands, and their regulation can be disrupted by various physiological and pathological conditions, including viral infections (55, 56). Consistent with this mechanism, FATP2 mRNA expression was significantly elevated at 24 h p.i., and FATP2 protein was also significantly increased at 48 h p.i. in ZIKV-infected SH-SY5Y cells. These virus-induced FATP2 expression by hepatitis B virus (57) and dengue virus (32) was shown in previous studies. Prior evidence indicates that ZIKV induces PPARγ in SH-SY5Y cells (35), suggesting that the virus may drive FATP2 expression via a PPARγ-dependent pathway.

In the FATP2 silencing experiment, the FATP2 mRNA and protein expressions were only moderately suppressed. We have tried various optimizations for the FATP2 silencing but could not achieve a drastic silencing. It is possible that a complete silencing may not be compatible with cell survival. Nevertheless, our results showed that, even with a moderate level of FATP2 silencing (Fig. 5C), the viral replication could be significantly suppressed (Fig. 5E), and that FATP2 is crucial for ZIKV production in SH-SY5Y. The reduction of ZIKV by FATP2 siRNA was lower compared to the activity of lipofermata reflecting the low level of suppression by the siRNA. Nevertheless, it is also possible that lipofermata may have some off-target effects that provide additional antiviral activity, which may be the target for a further study.

SH-SY5Y cells have been widely used as a model for ZIKV infection because they exhibit an immature neuronal phenotype, resembling the neuronal progenitor cells infected by ZIKV in fetuses (34, 58, 59). The baseline expression of FATP2 in SH-SY5Y makes it suitable for testing the lipid uptake inhibition exerted by lipofermata. In the present study, the concentration required to inhibit lipid uptake was higher than that needed for viral inhibition, and no cytotoxicity was observed in the short-term lipid uptake assay. A possible explanation is that viral inhibition was assessed over a longer incubation, allowing cumulative effects that produced a more pronounced antiviral outcome. We subsequently quantified cellular lipids at the conclusion of the incubation period (48 h p.i.) by using TLC. ZIKV increased PC and PE similar to the previous study (12), and a similar increase by hepatitis B virus infection was also reported (60). It might benefit replication by increasing the replication complex structure, which is generated from ER (15). Lipofermata decreased PC in ZIKV-infected SH-SY5Y. These might result in impairment of ZIKV replication by decreasing the replication complex. ER-associated degradation and translocation were associated with neural progenitor cells in genome-wide CRISPR screening (61). ER stress could trigger apoptosis and alter phospholipid composition leading to lipotoxicity (62). In addition, apoptosis could be triggered by PC in the adipocytes (3T3-L1) study (63). In this study, lipofermata decreased the ZIKV-induced extrinsic apoptosis pathway via caspases 3/7 and 8. Apoptosis in neural progenitor cells is linked to abnormal human brain development and microcephaly (29). Consequently, lipofermata may diminish the pathogenicity in neonates after maternal ZIKV infection. However, caspase 9 was not detected in our experiment.

In addition, prior lipidomic analysis of human serum samples revealed increased plasmalogen levels in ZIKV-infected individuals. Plasmalogens are phospholipids present in mammalian cell membranes, including those of neural cells. The synthesis of plasmalogens requires peroxisomes, which serve as vital sites for ZIKV replication. This increase in plasmalogens supports the concept that ZIKV replication is intricately linked to human lipid metabolism (64).

Previous research has shown that fatty acids acquired via FATP2 are destined for phosphatidylinositol synthesis (28). Phosphatidylinositol phosphates are critical for recruiting both viral and host proteins to replicative organelles, so inhibiting FATP2 could disrupt not only the overall lipid supply but also phosphatidylinositol synthesis, thereby impairing viral replication.

Additionally, recent lipidomic analyses have demonstrated the importance of fatty acids in ZIKV replication. Suppressing sphingolipid biosynthesis was found to reduce ZIKV infection, implying a critical role for sphingolipid metabolism (65). Because palmitic acid serves as a sphingolipid precursor, lipofermata-mediated fatty acid uptake inhibition may decrease sphingolipid production and ultimately disrupt ZIKV’s replication cycle. However, some lipid type was selected to observe based on previous study. A high-throughput approach for cellular lipid profiling, such as liquid chromatography-mass spectrometry, may encompass a broader lipid profile. Furthermore, cellular lipids seen microscopically may furnish credible proof to substantiate the drug’s efficacy.

Lipofermata was developed to inhibit fatty acid uptake, and it should be effective in type 2 diabetes and NAFLD (25). Lipofermata inhibited cancer cells, including bladder cancer cells (66) and melanoma (67). Given the lipid uptake inhibition of lipofermata and the necessity of lipids for viral life cycles, lipofermata was repurposed for viral studies, which reduced oxidative stress and inflammation resulting from lipid accumulation mediated by the hepatitis B virus in human hepatoma cell lines (57). In a recent study, lipofermata also inhibited dengue virus serotype 1 and 2 (32). Our results coincide with earlier studies, indicating an expansion of the viral inhibitory action of lipofermata on certain cell targets. In addition, the step of lipofermata inhibition in the ZIKV life cycle should be clarified further.

Notably, SH-SY5Y cells were not differentiated into mature neurons in this study. Previous work showed that differentiating SH-SY5Y with retinoic acid increases infectivity of the ZIKV prototype MR766 strain (first isolated in 1947 in Uganda) without altering the expression of the candidate ZIKV receptor Axl (36). Such differentiated SH-SY5Y cells may help elucidate neuropathogenesis in the developing or mature central nervous system. However, ZIKV primarily infects neuronal progenitor cells, which are not fully mature (30). Thus, undifferentiated SH-SY5Y cells more closely resemble neuronal progenitor cells and serve as a suitable model for ZIKV infection. However, the Warburg effect in SH-SY5Y should be a concern, especially in lipid metabolism (68). The effect of lipofermata between primary cells and cell lines may differ. Lipofermata activity and FATP2 function in ZIKV infection should be further tested in primary neuronal cells, organelles, or animal models.

In this study, undifferentiated SH-SY5Y cells yielded robust ZIKV production (approximately 10^6^ FFU/mL at an MOI of 5), which allowed evaluation of lipofermata’s inhibitory effect. Nonetheless, data on potential differences in FATP2 mRNA and protein expression between differentiated and undifferentiated SH-SY5Y cells are currently lacking.

For our study, we used a ZIKV clinical isolate from a Thai patient that was confirmed by genetic and antigenic characterization. Our findings offer the first evidence that ZIKV infection upregulates FATP2 expression and that inhibiting FATP2-mediated lipid uptake disrupts ZIKV replication in both imHC and SH-SY5Y cell lines. Lipofermata also reduced ZIKV-induced apoptosis, which may reduce microcephaly in newborns. In addition, FATP2 is essential for ZIKV from FATP2 silencing experiment. Given the high level of FATP2 expression in placental tissue, lipofermata might also exert anti-ZIKV effects in the placenta. Because ZIKV targets the liver, brain, and placenta and causes significant pathology in these organs, the anti-ZIKV activity of lipofermata appears highly relevant *in vivo*. However, data on whether lipofermata can pass the blood-brain barrier are still lacking. Nevertheless, additional studies are warranted to further evaluate and develop this novel antiviral strategy.

### Conclusion

Lipofermata, an inhibitor of FATP2, effectively inhibited ZIKV in imHC and SH-SY5Y cell lines. It also blocked fatty acid uptake in SH-SY5Y, suggesting that lipofermata suppresses ZIKV production and ZIKV-induced apoptosis via FATP2 inhibition. Notably, this study provides the first evidence that FATP2 is important for ZIKV life cycle. Moreover, ZIKV increases FATP2 mRNA and protein expression, which may facilitate the viral production.
